# Polyetheretherketone (PEEK) in Cranial Vault Reconstruction: A Review of Alloplastic Materials, Clinical Performance, and Institutional Experience

**DOI:** 10.3390/bioengineering13050567

**Published:** 2026-05-16

**Authors:** Christine F. Johansen, Shai M. Rozen

**Affiliations:** Department of Plastic Surgery, University of Texas Southwestern Medical Center, Dallas, TX 75390, USA; christine.johansen@utsouthwestern.edu

**Keywords:** cranial vault reconstruction, cranioplasty, polyetheretherketone (PEEK), alloplastic reconstruction

## Abstract

Cranial vault reconstruction is a critical component of care following trauma, decompressive craniectomy, infection, or oncologic resection. Large and complex cranial defects present unique reconstructive challenges, as autologous reconstruction may be limited by donor availability, contour mismatch, and long-term resorption. Consequently, alloplastic materials have become central to contemporary cranioplasty. Advances in imaging, computer-aided design/manufacturing (CAD/CAM), and three-dimensional workflows have enabled increasingly accurate patient-specific reconstruction with improved operative efficiency. This narrative perspective summarizes the evolution and clinical application of commonly used alloplastic materials in cranial vault reconstruction, including titanium, polymethylmethacrylate (PMMA), and hydroxyapatite (HA), with a focused discussion of polyetheretherketone (PEEK). We discuss the design rationale, material properties, and clinical performance of PEEK, emphasizing bone-mimicking elasticity, radiolucency, and compatibility with CAD/CAM workflows. Institutional experience is integrated to contextualize durability, aesthetic restoration, neurologic improvement, infection management, and technical considerations that influence outcomes. Overall, PEEK represents a versatile option for large and complex cranial reconstruction by combining favorable mechanical performance with patient-specific design capability. Continued advances in biomaterials and surgical planning, coupled with rigorous long-term outcome evaluation, will further refine material selection and optimize reconstructive outcomes in cranial vault reconstruction.

## 1. Introduction

Cranioplasty is performed to restore the integrity of the cranial vault following loss of native bone due to trauma, infection, tumor resection, congenital deformity, or decompressive craniectomy. In modern practice, cranial reconstruction serves purposes that extend beyond structural coverage alone. Restoration of a stable cranial contour contributes to cerebral protection, normalization of intracranial dynamics, and aesthetic outcomes [1,2,3,4,5,6]. In patients with large or complex defects, delayed or absent reconstruction may be associated with neurologic deterioration, including post-craniectomy syndromes characterized by headaches, cognitive changes, and motor impairment [2,4]. These considerations highlight the importance of durable and anatomically precise cranial reconstruction.

Defect size and local tissue quality are central determinants of reconstructive strategy. While smaller defects may be addressed with autologous bone, larger defects, often exceeding 20–25 cm^2^, are limited by donor availability, difficulty achieving durable contour, and risk of long-term resorption [2,7,8]. Patients with prior radiation, infection, or complex traumatic injuries are also poor candidates for autologous reconstruction. In these settings, alloplastic implants provide a consistent and structurally durable alternative [9,10].

Advances in material science, imaging, and computer-aided design/manufacturing (CAD/CAM) have shifted cranial reconstruction toward patient-specific synthetic biomaterials capable of accurately restoring complex cranial contour [11,12,13,14]. These technologies reduce intraoperative contouring while improving implant fit and surgical efficiency. Among available alloplastic materials, polyetheretherketone (PEEK) has gained increasing adoption due to its favorable mechanical properties, radiolucency, capacity for limited intraoperative modification, and potential for reuse in the setting of post-operative implant infection, a common complication of cranioplasty [15,16,17,18].

Historically, cranial reconstruction relied on metals, acrylics, and ceramics, which provided basic structural coverage but were limited by suboptimal biocompatibility, contouring challenges, and high complication rates [19,20,21]. Contemporary alloplastic materials are now broadly classified as metallic, acrylic, ceramic, or polymer-based implants. Selection requires balancing mechanical performance, elasticity, radiographic compatibility, infection risk, intraoperative modifiability, cost, and suitability for advanced manufacturing workflows [22]. This perspective review outlines the use of alloplastic materials in cranial vault reconstruction drawing emphasis on our long-term institutional experience using PEEK implants in cranioplasty.

## 2. Common Alloplastic Materials Used in Cranioplasty

### 2.1. Titanium

Titanium has been widely used in cranial reconstruction due to its strength, durability, and biocompatibility. It is commonly fabricated as mesh constructs or patient-specific plates, provides reliable structural support at it is osteointegrative, biocompatible, and easy to shape intraoperatively. However, it is not osteoconductive, and the radiopaque nature of titanium can disrupt postoperative surveillance. Its thermoconductive nature may cause paresthesia of the scalp and in some cases has caused skin burns over the implantation site [21,23,24,25,26]. Reported infection rates following titanium cranioplasty vary across series. In a recent systematic review of custom-made heterologous cranioplasties, the pooled infection rate for titanium implants was approximately 8%, with explantation required in fewer than 4% of cases; however, studies involving titanium tended to have shorter follow-up durations, potentially underestimating late-onset infection [27]. These limitations have driven continued investigation into alternative materials for large or complex cranial defects [21,28,29,30,31].

### 2.2. Polymethylmethacrylate (PMMA)

Polymethylmethacrylate (PMMA), formed through polymerization of methyl methacrylate (MMA), has been widely used in cranioplasty due to its radiolucency, favorable compressive strength, and capacity for intraoperative molding [22,32,33]. PMMA provides immediate defect coverage and can be fabricated using three-dimensional printing techniques for improved preoperative customization. However, PMMA lacks osteointegration, contributing to implant fracture, fragmentation, and higher long-term failure rates, particularly in large cranial defects [24]. Among commonly used alloplastic materials, PMMA has been associated with the highest reported infection rates. A large systematic review reported a pooled infection rate of approximately 15% following PMMA cranioplasty, with explantation required in over 6% of cases. Notably, PMMA studies tended to include longer follow-up durations, lending support to the robustness of these estimates [34,35]. As a result, the role of PMMA in contemporary cranial vault reconstruction has declined in favor of newer alloplastic materials with improved long-term clinical performance [20,22,27,34].

### 2.3. Hydroxyapatite (HA)

Hydroxyapatite is a bioactive calcium phosphate ceramic that constitutes the mineral component of native bone and is both biocompatible, osteointegrative, osteoconductive, allowing for partial integration with surrounding tissue [36,37,38]. However, hydroxyapatite provides limited mechanical strength and is prone to fracture, particularly during implantation or following trauma. Hydroxyapatite cranioplasty has demonstrated relatively lower reported infection rates in the literature, with pooled data suggesting infection rates of approximately 6–7% and explantation required in approximately 5% of cases which may be due to decreased biofilm production although studies in cranioplasty specifically have not been explored [27,39]. These findings are supported by several studies with extended follow-up; however, mechanical fragility continues to limit its use in larger defects [27,40]. As a result, hydroxyapatite is generally reserved for smaller cranial defects, restricting its applicability in extensive cranial vault reconstruction [27,40,41].

## 3. Polyetheretherketone (PEEK) in Cranial Vault Reconstruction

### 3.1. PEEK Material Properties and Design Rationale

Polyetheretherketone (PEEK) is a high-performance thermoplastic polymer whose mechanical properties closely approximate those of cortical bone. Its elastic modulus more closely matches native cranial bone than metallic implants, allowing for effective load distribution while minimizing stress shielding at the bone–implant interface. This bone-mimicking elasticity contributes to durable structural support and has made PEEK particularly well suited for reconstruction of large or complex cranial defects [22,42].

A key advantage of PEEK is its radiolucency and compatibility with postoperative surveillance. Unlike metallic implants, PEEK does not generate imaging artifacts, allowing for long-term assessment following cranioplasty [43,44]. Additionally, its thermal properties allow it to withstand radiation [45]. These imaging characteristics are especially advantageous in patients requiring ongoing neurologic monitoring.

PEEK is made with computer-aided design and manufacturing (CAD/CAM) workflows [5]. Patient-specific implants can be created using high-resolution preoperative imaging to achieve accurate restoration of cranial contour, even in complex cases [46,47,48]. This precision allows for improved aesthetic outcomes, decreased intraoperative contouring, and limited intraoperative modification [5,31].

An additional distinguishing feature of PEEK is its ability to be explanted, sterilized, and reused when appropriate [16]. This characteristic is particularly relevant in the setting of postoperative infection, where implant removal may be required. Reported infection rates following PEEK cranioplasty are intermediate relative to other alloplastic materials. In a systematic review of 379 PEEK implants, the pooled infection rate was approximately 11%, with explantation required in fewer than 4% of cases [27]. A recent meta-analysis and meta-regression of 1490 patients confirmed that PEEK demonstrated lower pooled all-cause complications (18.5%) and failures (6.3%; defined as explantation or reoperation requiring implant replacement) compared to both titanium (26.1% complications, 11.4% failures) and PMMA (28.4% complications, 12.7% failures). Notably, PEEK was the only alloplastic material that did not independently predict higher all-cause failures compared to autologous bone on multivariate analysis, though failures secondary to infection remained elevated across all alloplastic subtypes [49]. Interpretation of these findings is limited by variability in etiology, patient presentation and treatment history, follow-up duration, and study design, underscoring the importance of long-term institutional data when evaluating PEEK performance [27]. The capacity for reimplantation following resterilization, albeit experimental and not of standard practice, may offer an additional advantage over other alloplastic materials in the future and may reduce morbidity and cost in appropriately selected patients. However, it is important to note the risk of infection with reimplantation [11,44,50,51].

Additionally, PEEK implants carry a higher upfront cost compared to other alloplastic materials, averaging approximately $20,000 USD, though this is partially offset by elimination of intraoperative molding time and lower long-term complication rates [52].

### 3.2. Clinical Performance of PEEK

Clinical outcomes following PEEK cranioplasty have been consistently favorable. Reported cosmetic satisfaction rates range from 84–100%, reflecting the ability of patient-specific implants to restore cranial symmetry and contour, particularly in anterior and temporoparietal defects [14,16]. Importantly, these aesthetic outcomes are achieved without compromising structural integrity, reinforcing the dual functional and cosmetic role of PEEK in cranial vault reconstruction.

Management of infection remains a central consideration in cranioplasty [9,27]. While infection can occur with any alloplastic material, PEEK implants may be successfully managed with explantation and delayed reimplantation following adequate infection control. The ability to reuse the original patient-specific implant after sterilization represents a distinct advantage and has influenced reconstructive decision-making in high-risk and revision settings.

### 3.3. Surgical Considerations and Failure Modes

Successful PEEK cranioplasty depends on meticulous surgical planning and execution. Adequate soft tissue coverage is critical, as implant exposure remains one of the most common failure mechanisms. Tension-free closure, preservation of well-vascularized tissue, and selective use of adjunctive reconstructive techniques are essential, particularly in patients with prior radiation, infection, or multiple previous operations.

The timing of preoperative imaging relative to implant fabrication is also important. Delays between imaging and surgery may allow remodeling of defect margins, increasing the likelihood of intraoperative mismatch. Although PEEK permits limited intraoperative modification these may lead to marginal weakening, particulate contamination, and implant instability. Updated imaging within an appropriate preoperative window improves implant fit and reduces the need for adjustment. Failure of PEEK cranioplasty most commonly results from soft tissue compromise or infection rather than intrinsic material failure, highlighting the importance of surgical technique and patient selection.

## 4. Discussion

### 4.1. Institutional Experience

Our institutional experience with patient-specific PEEK cranioplasty includes both early revision cases and long-term follow-up cohorts, reflecting the complexity of patients undergoing secondary cranial reconstruction. In the initial series, 19 patients underwent 22 PEEK cranioplasty procedures, with a mean interval of 57.7 months between the initial cranial injury and loss of the primary cranioplasty. Traumatic cases demonstrated earlier loss of primary cranioplasty from time of injury, 4 months, underscoring heterogeneity in reconstructive timelines. Following loss of the primary reconstruction, the mean interval to PEEK cranioplasty was 11.8 months for infection-related cases and 12.2 months following trauma, whereas reconstructions for cosmetic or functional indications occurred earlier.

Operative management was relatively consistent across cases. Drains were placed in 11 of 22 procedures, and advanced soft tissue techniques were required in a subset, including adjacent tissue transfer in four cases and free tissue transfer in one. Intraoperative modification of the implant was required in four procedures: a vascular tumor abutting the defect, shortening to avoid nasal mucosa exposure following a difficult dissection, reshaping to afford lock-and-key fixation, and accommodation of interval bony growth on a CT obtained 14 months preoperatively. Reoperation following PEEK cranioplasty was uncommon, occurring in three patients and unrelated to implant fracture or intrinsic material failure.

**Figure 1:**
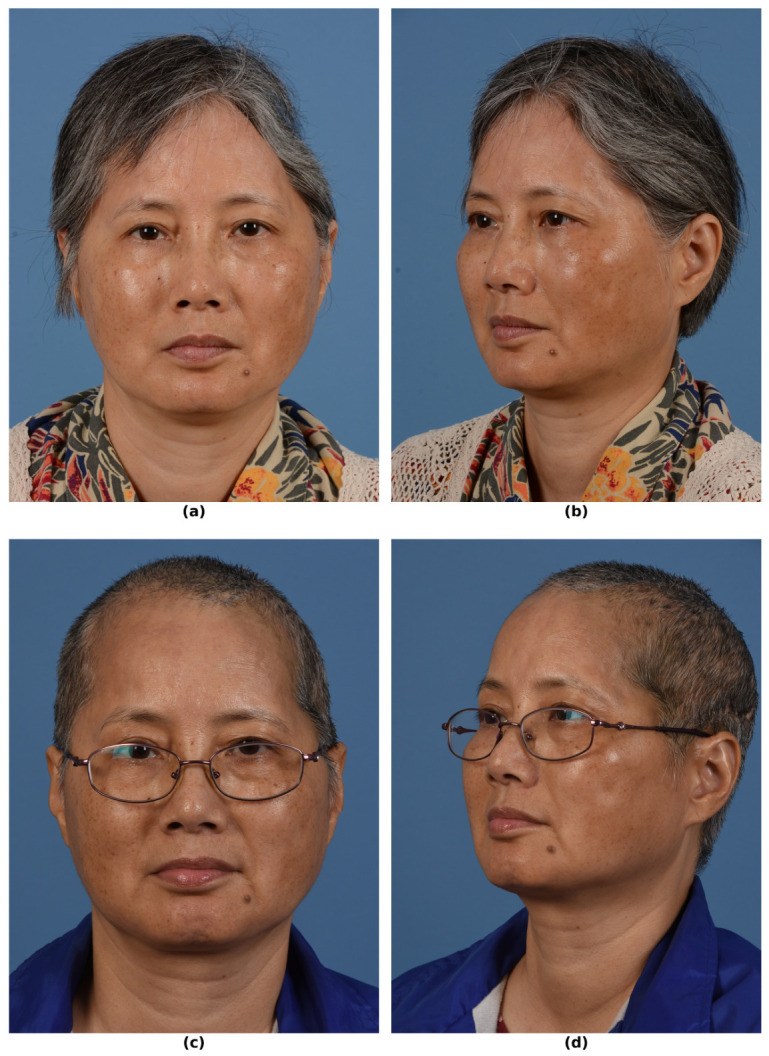
Preoperative and postoperative clinical photographs of a 59-year-old woman with a left frontotemporoparietal cranial defect following multiple decompressive craniectomies complicated by intracranial infections. She later had bony flaps which were then complicated by osteomyelitis and syndrome of trephined resulting in removal of the bone flap and closure with an adjacent tissue transfer. (**a**) Frontal photograph obtained approximately 9 months following bone flap removal and 2 months prior to definitive reconstruction, demonstrating a large cranial defect after removal of the infected bone flap. (**b**) Left oblique preoperative photo. (**c**) Photograph obtained approximately 15 months following delayed cranioplasty with a PEEK implant, showing restoration of cranial contour after prolonged infection resolution and soft-tissue healing. (**d**) Left oblique postoperative photo. (Used with permission of Shai M. Rozen, MD for Medical Education and Research. All rights reserved).

**Figure 2:**
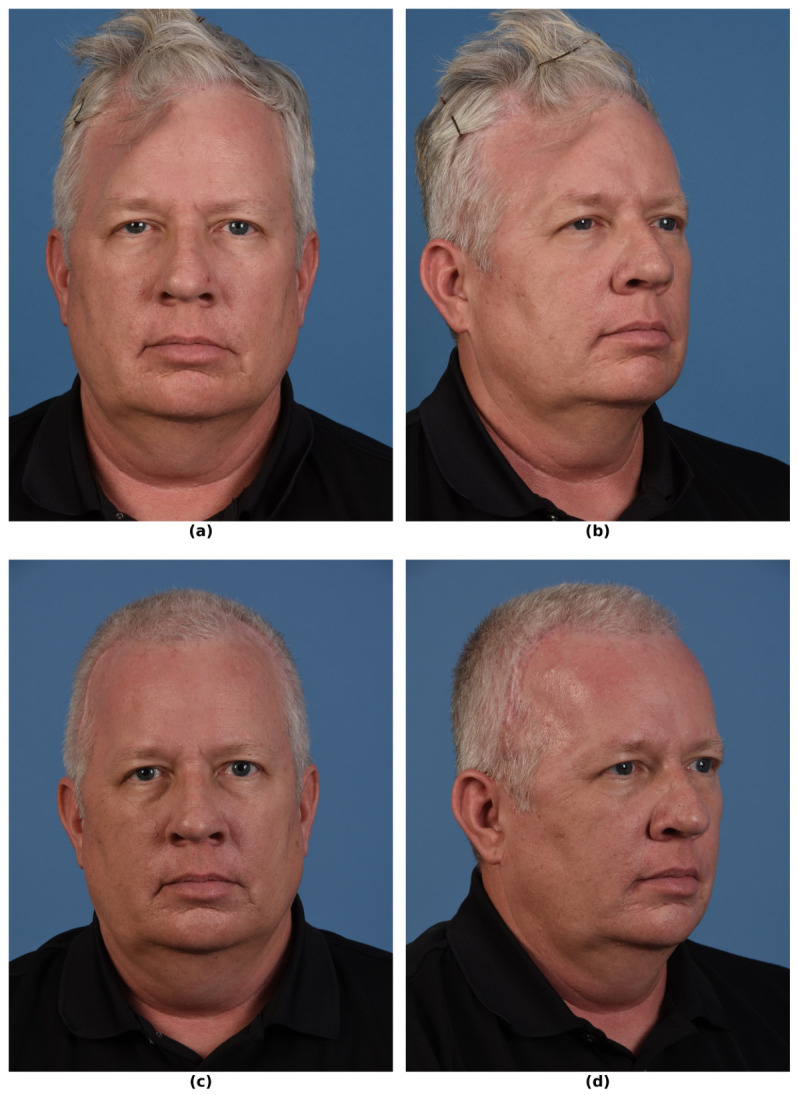
Preoperative and postoperative clinical photos of a 56-year-old man with a complex right frontotemporoparietal cranial defect following AVM surgery complicated by wound opening and osteomyelitis of the bone flap. He then received a PMMA implant and subsequent titanium mesh both complicated by open wounds. (**a**) Preoperative photograph obtained approximately 1 month prior to reconstruction and after removal of prior PMMA and titanium mesh implants. (**b**) Right oblique preoperative photo. (**c**) Photograph obtained approximately 1 month following cranioplasty with PEEK implant after prolonged debridement and infection control, illustrating stable soft-tissue coverage and restoration of cranial contour. (**d**) Right oblique postoperative photo. (Used with permission of Shai M. Rozen, MD for Medical Education and Research. All rights reserved).

**Figure 3:**
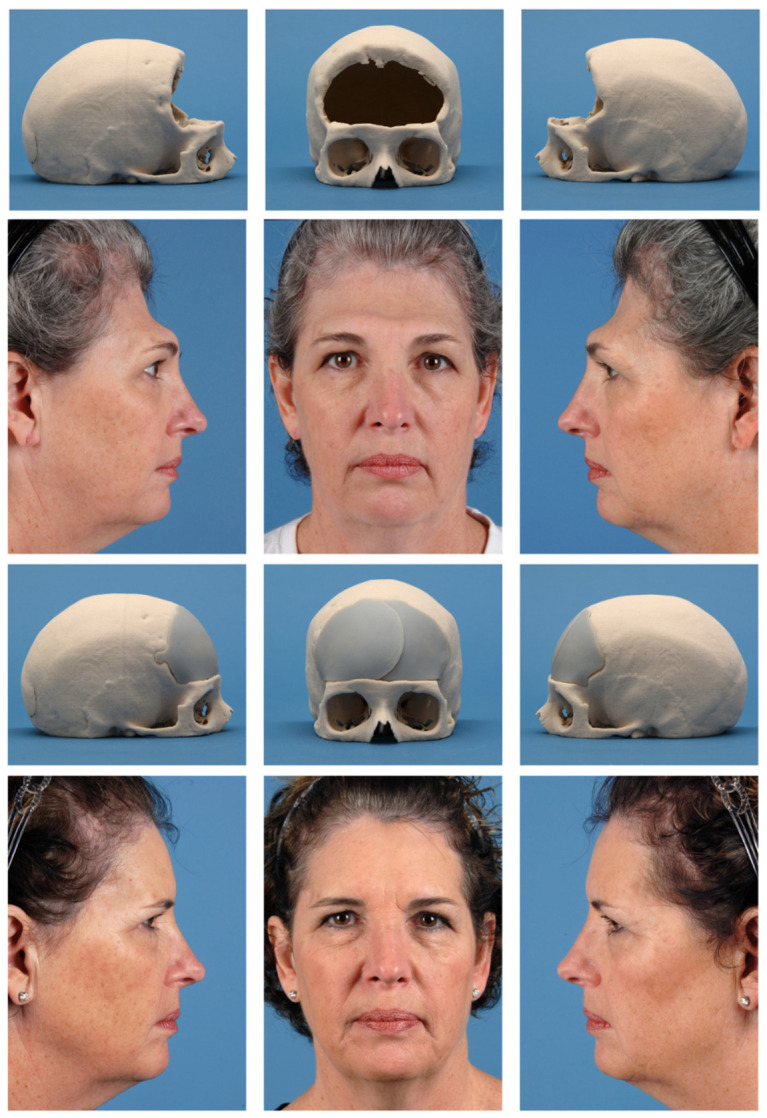
Preoperative and postoperative clinical and skull photos of a 55-year-old female patient with a history of craniotomy for meningioma resection complicated by autologous bone flap infection, resulting in a bilateral frontal cranial defect. Top row: Preoperative physical skull model demonstrating the extent of the bilateral frontal defect, obtained approximately 9 months following the initial craniotomy. Second row: Corresponding preoperative clinical photographs obtained approximately 1 month prior to reconstruction, shown in left lateral, frontal, and right lateral views. Third row: Postoperative physical skull model demonstrating placement of a patient-specific PEEK implant with restoration of frontal contour. Bottom row: Corresponding postoperative clinical photographs obtained approximately 25 months following reconstruction. (Used with permission of Shai M. Rozen, MD for Medical Education and Research. All rights reserved).

Long-term follow-up further reinforced the durability of PEEK cranioplasty. In a subsequent cohort of 22 patients followed for a mean of 83.45 months, patients had undergone an average of 2.95 prior cranial procedures before definitive PEEK reconstruction. Mean implant size was 180.43 cm^2^, and four patients required two-piece implants. Postoperative complications were infrequent and included one self-resolving subgaleal fluid collection, one hematoma, and three infections requiring explantation. One patient who required explantation secondary to infection after traumatic wound breakdown from a golf injury underwent resterilization and reimplantation of the original PEEK implant 14 months after the initial event, with no complications at 15-year follow-up. Although explantation with resterilization and reimplantation is feasible, it should be performed in select patients with proper soft tissue coverage and minimal changes in defect size and shape (Figure 1, Figure 2 and Figure 3).

Functional outcomes extended beyond structural reconstruction. In this long-term cohort, four of five patients with a preoperative seizure history reported postoperative improvement, and all patients presenting with symptoms consistent with the syndrome of the trephined experienced neurologic improvement. At extended follow-up, the majority of PEEK implants continued to provide durable cranial protection and sustained symptom relief, supporting the role of patient-specific PEEK cranioplasty as a reliable reconstructive option with both structural and physiologic benefits.

### 4.2. Limitations and Future Directions

Despite the favorable material properties and clinical performance associated with polyetheretherketone (PEEK) cranioplasty, several limitations warrant consideration. Much of the existing evidence is derived from retrospective series and single-institution experiences, introducing variability in patient selection, defect characteristics, and reconstructive complexity. In addition, long-term comparative data evaluating PEEK relative to other contemporary alloplastic materials remain limited, particularly with respect to functional neurologic outcomes and cost-effectiveness. Importantly, reported infection and explantation rates across materials are strongly influenced by etiology, follow-up duration, defect size, soft tissue condition, and radiation history and direct comparisons should be interpreted cautiously in the absence of standardized reporting thresholds.

From a material standpoint, ongoing investigation into surface modification, antimicrobial strategies, and hybrid implant constructs may further improve biocompatibility and reduce infection risk. Current literature has identified only trends toward increased infection with smooth implants representing a promising direction in implant surface [53]. Concurrent advances in imaging, virtual surgical planning, and manufacturing workflows are also likely to continue refining implant accuracy and operative efficiency. This includes advances in bone tissue engineering, such as mineralized collagen–glycosaminoglycan scaffolds that induce calvarial regeneration with exogenous cells or growth factors [54]. Future studies incorporating standardized outcome measures, prospective design, and longer-term follow-up will be essential to more clearly define optimal indications for PEEK cranioplasty and to guide material selection in increasingly complex cranial reconstruction.

## 5. Conclusions

Alloplastic materials play a central role in modern cranial vault reconstruction, particularly for large or complex defects in which autologous reconstruction is limited. Among available alloplastic materials, titanium offers high strength but limited postoperative surveillance and has a thermoconductive nature; PMMA provides intraoperative flexibility at the cost of higher infection rates; hydroxyapatite affords bioactivity but has limited strength. Polyetheretherketone (PEEK) offers a distinctive combination of bone-mimicking mechanical properties, radiolucency, patient-specific design capability, and intraoperative versatility. Clinical experience supports its durability, reliable restoration of cranial contour, and utility in complex primary and revision settings, including cases complicated by prior infection.

Although no single material is universally ideal, PEEK has emerged as a leading option in contemporary cranioplasty due to its balanced material performance and adaptability to demanding reconstructive scenarios. Ongoing advances in biomaterials, imaging, and surgical planning, together with continued long-term outcome evaluation, will further refine its indications and guide future innovation in cranial reconstruction.

## Data Availability

No new data were created or analyzed in this study. Data sharing is not applicable to this article.

## Abbreviations

The following abbreviations are used in this manuscript:

PEEK	Polyetheretherketone
PMMA	Polymethylmethacrylate
HA	Hydroxyapatite
